# Context-Dependent Effect of Reverberation on Material Perception from Impact Sound

**DOI:** 10.1038/s41598-017-16651-4

**Published:** 2017-11-28

**Authors:** Takuya Koumura, Shigeto Furukawa

**Affiliations:** NTT Communication Science Laboratories, 3-1, Morinosato Wakamiya Atsugi-shi, Kanagawa, 243-0198 Japan

## Abstract

Our hearing is usually robust against reverberation. This study asked how such robustness to daily sound is realized, and what kinds of acoustic cues contribute to the robustness. We focused on the perception of materials based on impact sounds, which is a common daily experience, and for which the responsible acoustic features have already been identified in the absence of reverberation. In our experiment, we instructed the participants to identify materials from impact sounds with and without reverberation. The imposition of reverberation did not alter the average responses across participants to perceived materials. However, an analysis of each participant revealed the significant effect of reverberation with response patterns varying among participants. The effect depended on the context of the stimulus presentation, namely it was smaller for a constant reverberation than when the reverberation varied presentation by presentation. The context modified the relative contribution of the spectral features of the sounds to material identification, while no consistent change across participants was observed as regards the temporal features. Although the detailed results varied greatly among the participants, these results suggest that a mechanism exists in the auditory system that compensates for reverberation based on adaptation to the spectral features of reverberant sound.

## Introduction

**Figure 1:**
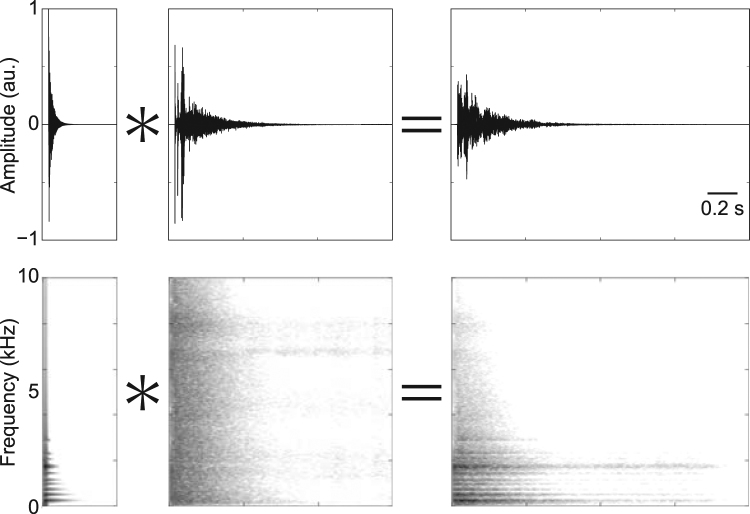
An impact sound with and without reverberation. Waveforms (top) and spectrograms (bottom) of a non-reverberant impact sound of wood (left) convolved with a room impulse response (middle) resulting in a reverberant sound (right). Reverberation alters both the temporal and spectral characteristics of the sound. As with the example shown in the figure, reverberation generally lengthens the decay time of a sound. The degree and pattern of the alteration depend on the RIR.

Sound emitted from a source is usually modified by the surroundings before reaching our ears^1^. One such example of modification is room reverberation, which is the result of sound reflecting on various objects in the immediate environment^2^ (Fig. 1). The process of reverberation is simulated by convolution of the original sound from a source with a room impulse response (RIR). This study concerns the way in which the reverberation affects auditory perception and explores the strategy used by the auditory system to extract reliable information about a sound source in the presence of reverberation.

Reverberation is known to degrade the quality of speech and therefore the performance of speech recognition^3,4^. When a target is a relatively long sequence of signals as in speech, the persistence of the acoustic energy of preceding sounds makes subsequent signals less audible due to masking or interference^4^. Moreover, even when a target signal is presented in isolation, any alteration of acoustic cues as a result of an extended decay time or the spectral envelope being modulated by an RIR would affect the signal identification performance^5,6^. For example, Watkins showed that the presence of reverberation shifts the categorical boundary in consonant identification along a continuum between the English words “sir” and “stir”^6^. The continuum was formed by modulating the depth of the temporal envelope of the signal, and adding reverberation reduced the depth of the modulation.

In everyday life, the perception of speech and other sound sources is generally robust and stable regardless of diverse reverberation conditions, where no information about the RIR is explicitly provided. A priori knowledge of the statistics of natural reverberations can contribute to robustness^2,5^. A quantity of evidence also suggests that the auditory system compensates for the effects of reverberation based on some form of information about room reverberation obtained from the acoustic context in which a target signal is embedded^6–12^. For example, Brandewie and Zahorik have shown that the recognition accuracy for a speech superimposed with a noise masker improves when the target speech is preceded by another speech recorded in the same environment as the target speech^7^. Watkins showed that the effect of reverberation is reduced when the target word is embedded in longer speech with the same reverberation as the target word^6^. Although there is a counter argument stating that such observation is not due to compensation for reverberation but to interference by the carrier sentence^13^, these studies suggest that reverberation affects our perception in a context-dependent manner.

The evidence for the context-dependent effect of reverberation has been largely collected using speech signals and simple synthetic sounds such as tones and noises. However, speech perception is known to involve multiple acoustic cues that are often redundant and that interact with each other^14,15^, and simple synthetic stimuli do not induce behaviourally relevant perception in real listening situations.

The present study tries to solve these problems by focusing on material perception based on impact sounds. We encounter impact sounds frequently in our daily lives, and from them we can consciously or unconsciously glean information about objects or environments^16–18^: We can recognize, for example, the material of a floor (e.g., wood or stone) from the sounds made by footsteps. It is also advantageous that the acoustic features for material perception are relatively well characterized^19–22^. Namely, decay rate and spectrum profile have been suggested as important cues for material perception based on impact sounds. Aramaki *et al*. have investigated the relative contribution of the following 4 acoustic features to material perception: attack time, decay rate, spectral bandwidth, and spectral roughness^19^. The attack time and decay rate are temporal features computed from amplitude envelopes, and spectral bandwidth and spectral roughness are spectral features computed from spectra. Their conclusion is that the decay rate is especially important for material perception. The cue that made the second largest contribution was spectral roughness, which depends largely on spectral shape.

In a real situation, such features are expected to be altered by reverberation^2^. Generally, reverberation increases the decay time of a sound by various degrees depending on the frequency^5^. Consequently, material perception based on impact sound can be affected by reverberation, possibly in a context-dependent manner as in speech perception.

We conducted an experiment in which participants listened to various impact sounds and attempted to identify the material that generated the sounds. The sounds were presented in their original form or convolved with RIRs that simulated the reverberations of various rooms. We presented the stimuli in two different reverberation contexts. In one context, impact sounds with the same reverberation were presented repeatedly, thus simulating hearing various sounds in a single room (constant room condition). In another context, the reverberation types were varied for each sound (varying room condition). We compared the size of the reverberation effect between the two contexts. Given the assumption that the same kind of compensation mechanism exists as that previously shown for speech perception, we expected that the effect of reverberation would be smaller in the constant room condition. Furthermore, to find the acoustic features responsible for the context dependence, we evaluated the relative contributions of acoustic cues to material identification and compared them for the two contexts.

## Results

### Population analysis of material perception based on impact sound

In the experiment, the participants identified the material that produced the impact sounds as wood, metal, or glass. We used the impact sounds provided by Aramaki *et al*.^19^. The stimuli consisted of 15 impact sounds (5 wood, 5 metal, and 5 glass). Original material sounds are usually classified with little ambiguity by participants^19^. Therefore, sounds produced from ambiguous materials synthesized by morphing two sounds from different materials (e.g. wood and metal) were also included in the stimulus set^19^. Reverberation was simulated by convolving the impact sounds with an RIR from a database^23^. We simulated reverberation in 3 different rooms (rooms #1, #2, and #3) using 3 impulse responses (Supplementary Fig. 1, Supplementary Table 1).

**Figure 2:**
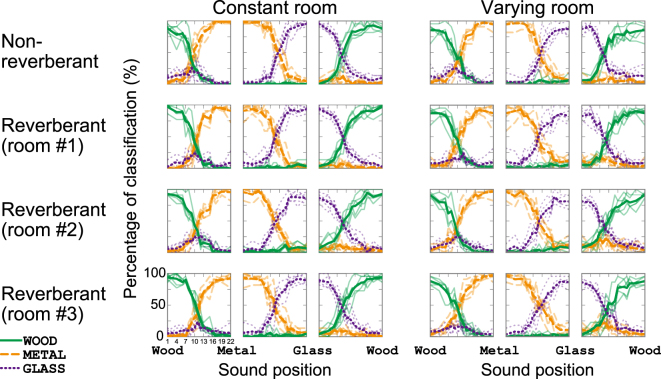
Population responses to impact sounds. Proportion of participants selecting each material (solid green line: wood, dashed orange line: metal, dotted purple line: glass) under constant (left) and varying (right) room conditions. From top to bottom: non-reverberant sounds, reverberant sounds in rooms #1, #2 and #3. The horizontal axis shows the sound positions in the sound continua between different materials. The dark thick lines indicate the average of the responses to all objects with the same material pairs. The light thin lines indicate the classification percentages in each object pair. As expected, the original sounds of wood, metal, and glass (left- or right-most sounds on the abscissa of each panel) were most frequently classified as wood, metal, and glass, respectively. In some cases, a small proportion of the participants classified intermediate sounds along the continuum in a category that did not represent either of the sound pair constituting the continuum. For example, the “glass” response (dotted purple) appeared for the wood-metal continuum (e.g., top left panel). Generally, the pattern of the responses was not markedly different among the reverberation types (compare panels vertically). The light thin lines in the top row are comparable with Fig. 2 of Aramaki *et al*.^19^.

The tendency of the population as regards material identification is summarized as the proportion of the participants who categorized a particular sound as a particular material (Fig. 2). The patterns of classification when reverberation was absent (Fig. 2, top left panels) were similar to those obtained in a previous study (comparable to Fig. 2 in Aramaki *et al*.^19^). We confirmed that the majority of participants gave responses that were associated with the original sound classes. For instance, 88% of the participants categorized wood sounds as wood (Fig. 2, top left panel). As the stimulus was morphed along the continuum towards metal sounds, the number of participants who selected wood gradually decreased and a growing number of participants started to identify the sound as metal. As also found in the previous study^19^, a small proportion of responses were observed that chose the third material class for sounds in the centre of the continuum. For example 25% of the participants selected glass for the intermediate sound between wood and metal.

Reverberation appeared to have little effect on the pattern of classification by the participant population. The classification percentages for the reverberant conditions (Fig. 2, second to fourth rows) were very similar to those for the non-reverberant condition (top row). Indeed, the correlation coefficients between the classification percentages for the non-reverberant conditions and those for the reverberant conditions were close to 1 (Table 1, second row in each cell; 99% bootstrap confidence intervals are shown in the square brackets).

**Table 1:** Comparison of pairs of population data with different reverberation types.

	Constant room condition			Varying room condition		
	Reverb.(room #1)	Reverb.(room #2)	Reverb.(room #3)	Reverb.(room #1)	Reverb.(room #2)	Reverb.(room #3)
Non- reverb.	0.154;0.95;[0.94, 0.96]	0.151;0.95;[0.94, 0.96]	0.164;0.94;[0.92, 0.95]	0.161;0.95;[0.94, 0.96]	0.160;0.95;[0.94, 0.96]	0.161;0.95;[0.93, 0.95]
Reverb. (room #1)	—	0.146;0.96;[0.95, 0.97]	0.151;0.96;[0.94, 0.96]	—	0.137;0.95;[0.94, 0.96]	0.142;0.95;[0.94, 0.96]
Reverb. (room #2)	—	—	0.146;0.96;[0.95, 0.97]	—	—	0.128;0.95;[0.94, 0.96]

Separated by semicolons in each cell: Hellinger distance, correlation coefficient, and 99% bootstrap confidence intervals of the correlation coefficient.

### Effect of reverberation on individuals

**Figure 3:**
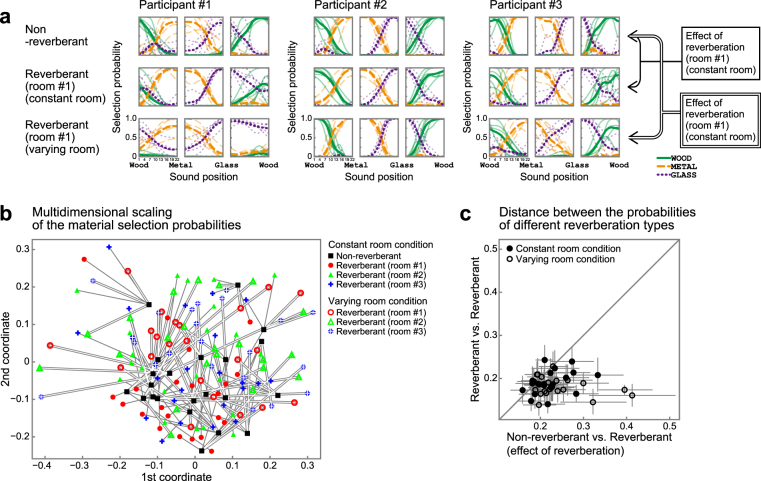
Material identification by individual participants. (**a**) Materials identified by 3 participants are shown as examples. With each participant, we show the probabilities of the selected materials for the non-reverberant sounds (top rows), the reverberant sounds in room #1 in the constant room condition (middle rows), and the varying room condition (bottom rows). Other conventions are the same as in Fig. 2. The functions were estimated with the kernel smoothing method as described in Materials and Methods. Generally, the psychometric functions differed markedly for non-reverberant and reverberant sounds (compare the panels vertically), indicating that material perception was affected by reverberation. The probabilities for the constant room and varying room conditions were also different, indicating the context dependence of the effect of reverberation. The effect of reverberation was defined by the distance between the probability for non-reverberant sounds and that for reverberant sounds, as illustrated on the right in the figure. The probability patterns varied among the participants, both for non-reverberant and reverberant sounds. (**b**) Multidimensional scaling of the material selection probabilities. Material selection probabilities under all conditions for all participants are plotted in the 2-dimensional space of the multidimensional scaling of the Hellinger distance. Black squares: responses to non-reverberant sounds, red circles: reverberant sounds in room #1, green triangles: in room #2, blue crosses: in room #3. Filled and open symbols represents the constant and varying room conditions, respectively. Dots for a single participant are connected with single (constant room condition) and double (varying room condition) lines. (**c**) Distance between the probabilities of different reverberation types. Distance between the responses to non-reverberant and the responses to reverberant sounds (abscissa) were larger than the distance between the pair of responses to reverberant sounds in different rooms (ordinate). Each dot represents a single participant. Filled circles: constant room condition, open circles: varying room condition. The error bars indicate the 95% bootstrap confidence intervals.

In contrast to the population profile, the response patterns varied among individuals, and more importantly, marked effects of reverberation were observed when the individual responses were examined. For each participant, we estimated the probability of material selection conditional on the stimulus identity (Fig. 3a, Supplementary Fig. 2). Figure 3a shows the responses of 3 participants as examples (see Supplementary Fig. 2 for the results of all participants). Note that the psychometric functions in the figure were derived by smoothing the actual data with a certain kernel (see Materials and Methods for details). Participant #1, for instance, responded “wood” less frequently for sounds in room #1 than for sounds without reverberation (compare the top and middle rows in Fig. 3a), suggesting that the perceived material changed as a result of the reverberation. Participant #2 appeared less affected by reverberation, and participant #3 responded “wood” less often and “glass” more often due to reverberation.

As can be seen in those examples, there was considerable variety in the patterns of the reverberation effects among participants. This is visualised by the multidimensional scaling of the material selection probabilities of all reverberation types in all participants (Fig. 3b). In the figure, responses for each reverberation type and each participant are represented as a single point, and for each participant, the points representing reverberant conditions (coloured points) are connected with lines to the point representing the non-reverberant condition (black points). The collective distribution of the points for the reverberant conditions appears to overlap the distribution for the non-reverberant condition, which is consistent with the earlier notion that on average the presence of reverberation has little effect on material judgment. Participant-by-participant inspections, however, reveal that the points for the reverberant conditions tended to deviate markedly from that for the non-reverberant condition (indicating the effect of reverberation), and there was no systematic trend across the participants in the directions of the deviations (indicating a variability in the pattern of the reverberation effect).

The response variation within each participant observed here reflects largely the effect of reverberation, and not mere measurement variability, as supported by the following analyses. We compared the following two quantities: the average distance between the responses to non-reverberant sounds and the responses to reverberant sounds (Fig. 3c, abscissa); and the average distance between the responses to reverberant sounds in one room and those in another room (Fig. 3c, ordinate). If the differences in the responses to the non-reverberant and reverberant sounds observed for an individual participant are dominated by response variability (or “measurement noise”), we can expect there to be no systematic difference between the two distance values. In fact, the analysis result contradicted this expectation. The distance of non-reverberant vs. reverberant sounds was larger than that of reverberant vs. reverberant sounds (Wilcoxon signed rank test, constant room condition: *p* = 0.0025, varying room condition: *p* = 0.00031). In other words, the 3 RIRs induced similar responses to each other, whereas they were significantly different from the responses to the non-reverberant sounds, indicating that the presence of reverberation was the major factor determining the material perception.

The apparently small effect of reverberation described in the previous section (Fig. 2) can be accounted for by the participant-dependent reverberation effects being cancelled out by averaging. Thus, the following analyses were conducted on an individual participant basis.

### Context-dependent effect of reverberation

**Figure 4:**
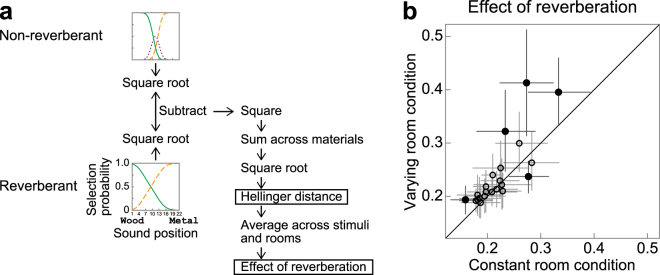
Size of reverberation effect. (**a**) Schematic to show how the reverberation effect was calculated. (**b**) Comparison of the sizes of the reverberation effects under the constant (horizontal axis) and varying (vertical axis) room conditions. Each circle represents the size of the effect (i.e., average Hellinger distance of the psychometric functions) for one participant. The error bars indicate the 95% bootstrap confidence intervals. Filled circles: dots with error bars not crossing the diagonal line, open circles: dots with error bars crossing the diagonal line. Although the overall effect varied among the participants, the size of the effect was generally greater under the varying room condition than that under the constant room condition (*p* < 0.05), indicating the context dependence of the effect of reverberation.

The main interest in the present study was the effect of presentation context on material perception in the presence of reverberation. Here, by “context” we mean whether the same room reverberation was presented in a block of trials (constant room condition) or varied trial by trial (varying room condition). We focused on the *size* of the reverberation effect, rather than examining the *pattern* of the effect in detail. The size of the reverberation effect for a given participant and context condition was quantified as the distance (namely the Hellinger distance; see Materials and Methods) between the psychometric function (probability of response versus impact sound) for the reverberation condition of interest and that for the non-reverberation condition (Fig. 4a). A greater distance indicates a greater effect of reverberation on identifying materials. The results for the three room reverberations (i.e., rooms #1, #2, and #3) were averaged. Figure 4b compares the distance for the varying room condition (ordinate) and that for the constant room condition (abscissa). Each point in the figure represents one participant, and the error bars indicate the 95% confidence intervals estimated by the bootstrap method. The points were generally above the diagonal line. The Wilcoxon signed rank test (a non-parametric statistical test, which is robust to the presence of outliers) indicates that the distance was generally smaller for the constant room condition than for the varying room condition (Fig. 4b; *p* = 0.011). This indicates that repeated presentation of the same reverberation reduced the effect of reverberation on the identification of materials. Moreover, the size of the effect appeared similar across the RIRs (Supplementary Fig. 3). Thus the observed effect of reverberation was not specific to certain RIRs, but was a general phenomenon across different RIRs.

However, the effect of the context was generally small compared with the within-participant variation of responses; for all but 5 participants, the confidence interval crossed the diagonal line. Four of them exhibited a statistically larger distance (i.e., the error bar crossing the diagonal line) for the varying room condition than that for the constant room condition, and one participant exhibited the opposite.

### Context effects on relative contributions of acoustic features

**Figure 5:**
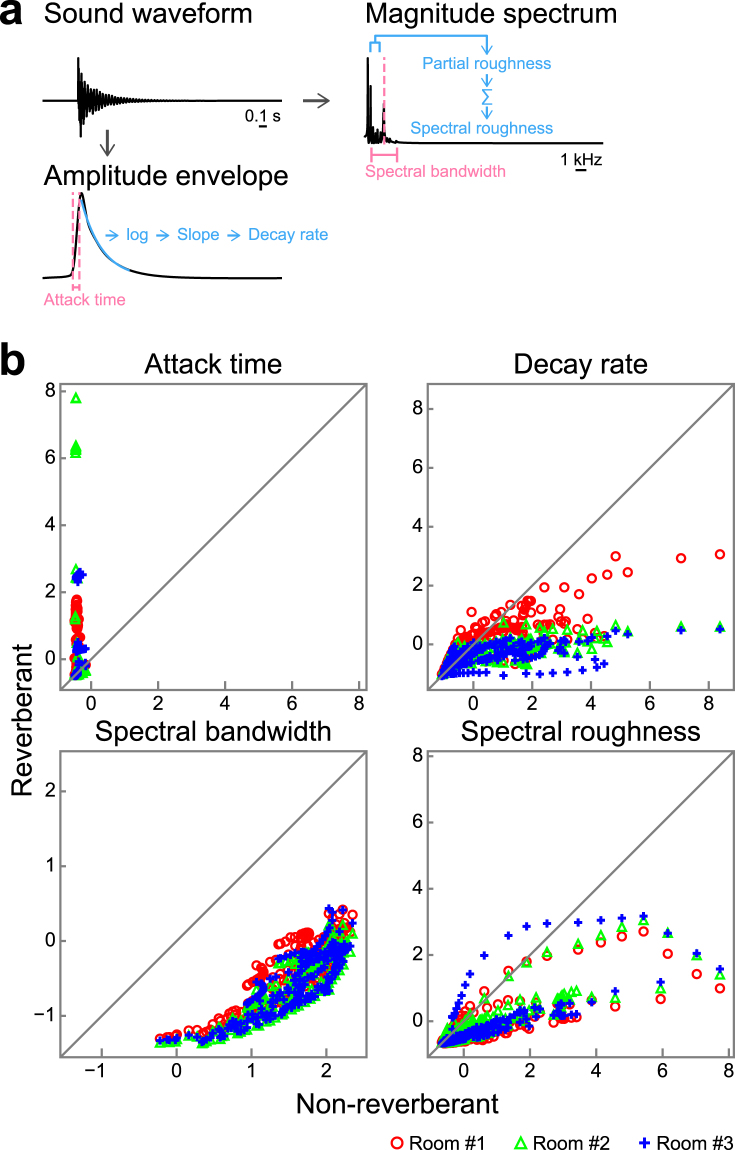
Acoustic features of non-reverberant and reverberant sounds. (**a**) Schematic showing how the acoustic features were calculated. An amplitude envelope and a magnitude spectrum were calculated from a sound waveform. The attack time and decay rate were calculated from the amplitude envelope, and the spectral bandwidth and spectral roughness were calculated from the magnitude spectrum. (**b**) Comparison of indices of acoustic features of non-reverberant sounds (horizontal axis) and reverberant sounds in room #1 (vertical axis, red circles), room #2 (green triangles), and room #3 (blue crosses). Each dot represents one sound. The indices are represented by z-scores. The attack time increased due to reverberation. The decay rate, spectral bandwidth, and spectral roughness decreased due to reverberation.

The previous study has shown that the decay rate provides the strongest clue to identifying materials from impact sound, followed by the spectral cues^19^. In the real situation, however, such features may be altered by reverberation. Indeed, we confirmed that reverberation changed both the temporal and spectral features of impact sounds (Fig. 5). As in the previous study^19^, we computed the following 4 acoustic features of the stimuli: attack time, decay rate, spectral bandwidth, and spectral roughness (Fig. 5a). Figure 5b compares the acoustic features of the sounds without reverberation and those in room #1. Changes of the features are clearly recognizable in the scatter plots. The attack time increased due to reverberation, and the decay rate decreased. Both spectral features decreased due to reverberation.

**Figure 6:**
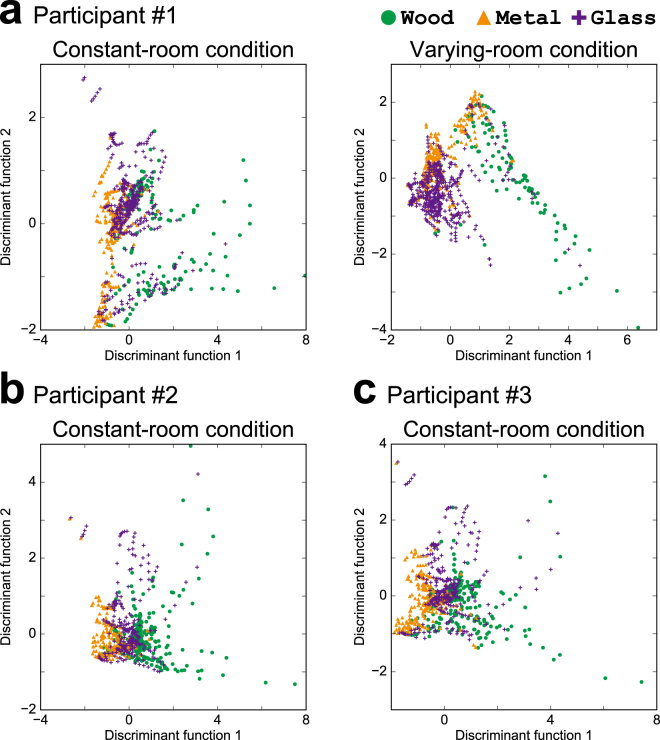
Subspaces in which selected materials are most separated. Two-dimensional subspaces computed by LDA with four acoustic features. Examples for 3 participants are shown (**a)** participant #1; (**b)** #2; (**c)** #3). For participant #1, plots in the constant room condition (left) and varying room condition (right) are shown. For participants #2 and #3, plots in the constant room condition are shown but those in the varying room condition are omitted. Each dot represents one sound. Colours and shapes indicate materials (green circles: wood, orange triangles: metal, purple crosses: glass). Selected materials appeared well separated by the linear combination of the acoustic features in both contexts.

**Figure 7:**
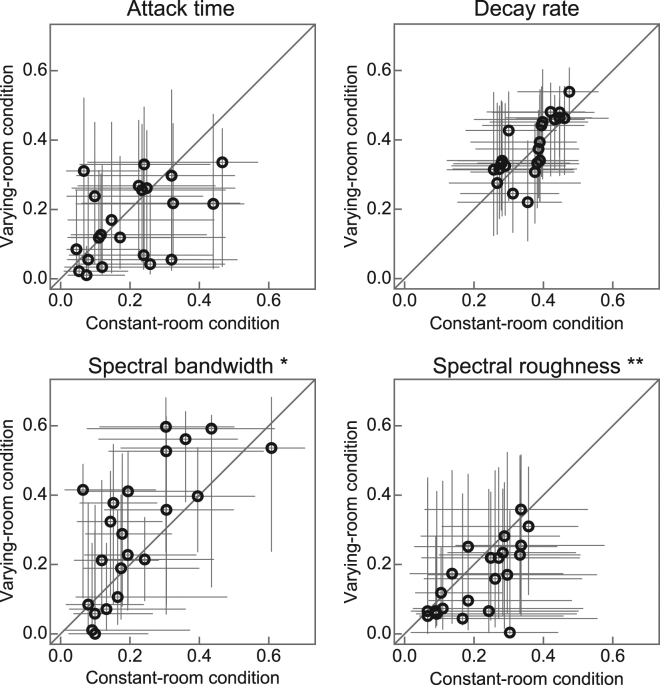
Contribution of acoustic features to material identification. Comparisons of the contributory weights on each acoustic feature under constant (horizontal axis) and varying (vertical axis) room conditions. Each panel represent one feature as indicated above the panel. Each circle represents one participant. The error bars indicate the 95% bootstrap confidence intervals. The contribution of the spectral bandwidth to material identification was larger under the varying room condition (*p* < 0.05), and that of spectral roughness was larger under the constant room condition (*p* < 0.01). The contributions of the attack time and decay rate did not differ statistically between the conditions (*p* > 0.1). **p < 0.01, *p < 0.05.

The observed context dependence of the effect of reverberation might be a result of the auditory system processing and/or combining of such features in a context dependent manner. To explore this possibility, we built a model that explains the participant’s response using a linear combination of the acoustic features of the stimuli (Fig. 6, Supplementary Discussion 1). Then, based on the model, we evaluated the contribution of individual features to the identification of materials by each participant and in each condition (Fig. 7).

The contribution of the spectral bandwidth to material identification was larger in the varying room condition than in the constant room condition (Wilcoxon signed rank test, *p* = 0.032), and the contribution of the spectral roughness was larger in the constant room condition (*p* = 0.0018). The attack time and decay rate did not differ with the conditions (attack time: *p* = 0.18, decay rate: *p* = 0.21). In other words, the contribution of the spectral features to material identification were context-dependent, but those of the temporal features were not.

## Discussion

### Adaptation to reverberation

We found that the effect of reverberation on material perception was smaller when sounds with a single reverberation were presented repeatedly (constant room condition) than when sounds with various reverberations were presented in random order (varying room condition) although the effects were varied greatly among participants and not all analyses showed a consistent effect across all participants. We also found that there were context-dependent changes in the contribution of the spectral features to material identification. To the best of our knowledge, this is the first time that the context-dependent effect of reverberation has been observed using naturalistic stimuli other than speech or simple synthetic sounds such as tones, noises, or clicks.

Our result suggests the existence of auditory mechanisms adaptive to reverberation. This finding is in line with previous studies, in which adaptation to reverberation has been shown using speech^6–9^, tones^11^, or noises^10–12^. In our daily life, once we enter a room, we continue to be exposed to sounds with almost the same reverberation, although sound sources can vary. It is possible that the auditory system takes advantage of this natural tendency to achieve robust material recognition in a reverberant environment even when the RIR is not given in an explicit form.

### Importance of spectral features

We confirmed that reverberation changed both the temporal and spectral features of impact sounds (Fig. 5b). The attack time increased due to reverberation, and the decay rate decreased, meaning that the sound was temporally smoothed due to reverberation. The attack times of some sounds were greatly elongated. This is because attack time was calculated based on the first peak in the amplitude envelope of the sound (see Materials and Methods for details). Amplitude envelopes of some sounds consisted of multiple peaks, and in some cases, the first peak disappeared due to reverberation, making the (original) second peak the reference for the attack time calculation.

It is known that the increase in decay time caused by reverberation is frequency-dependent. The amount of the increase tends to be largest around 1 kHz^5^, thus probably making the long-term power spectrum gain around 1 kHz. The spectral bandwidth decreased due to reverberation, meaning that the power of the sound concentrated around a certain frequency. The spectral roughness was reduced by reverberation, probably because reverberation introduced spectrally incoherent fluctuations^24,25^.

A previous study suggested that the decay rate is the feature that makes the biggest contribution to material identification, followed by the spectral features^19^. Intuitively the most prominent effect of reverberation on a sound is an increase in its decay time^2^, and thus we expected that adaptation to the context should be apparent in the temporal properties. Interestingly, the context-dependence of the relative contribution was observed for spectral features but not for temporal features. This finding does not necessarily contradict the results of the previous study indicating that the decay rate makes the largest contribution to material identification. Temporal features probably remain important for material recognition even under reverberation, and the features responsible for adaptation to reverberation are spectral features. In other words, although the decay rate could play a key role in material perception in and out of reverberation, in this study the context dependence of the cue was not consistent across the participants. It is premature to rule out the possibility that the contributions of the temporal features are context-dependent. There is a possibility that the contribution of the temporal features was dependent on the context, while the changes were not consistent across the participants and thus not detected as a population tendency (Fig. 7).

With a limited amount of experimental data, the present study focused on just 4 features because the previous study demonstrated that they are sufficient to describe material perception based on “dry” sounds^19^. There is, however, the possibility that the 4 features could not capture the whole nature of the impact sound with reverberation. Reverberation may distort various other features of the sound, on which the participant may rely. Strong conclusions should not be derived until future work identifies the essential features that account for material perception under reverberation.

### Individual differences in reverberation effect

Our results indicate that averaging responses over participants reduces the effect of reverberation that could be observed in the individual participants (Figs 2 and 3). This implies that strategies for conducting the material identification task may be different among individuals. In spite of the large variability among individuals, participants exhibited a tendency to adapt to reverberation. This suggests the universality of the adaptation to reverberation irrespective of individual strategies. Such insight might only be obtained using stimuli with substantial variability but might not be obtained using stimuli with small degrees of freedom as in most psychophysical studies.

### Moving beyond speech

Previous studies of speech perception in the presence of reverberation have suggested the existence of auditory mechanisms that adapt to presentation contexts to achieve robust perception in realistic environments^6–9^. The present study, which deals with impact sounds, marks an important stage in this series of studies, by suggesting that the scope can be extended from speech sounds to a general class of sounds. Recognizing the material of a sound source is also important for the survival of non-human animals. Behavioural or physiological animal studies using a similar paradigm to this study will elucidate neurophysiological mechanisms related to how the auditory system copes with reverberation^26–28^.

## Materials and Methods

### Participants

Twenty-two adults (16 females, ages between 21 and 46) participated in the experiment. All participants gave informed written consent before the experiments. All procedures were approved by the NTT Communication Science Laboratories Ethical Committee and conducted in accordance with the Declaration of Helsinki.

### Task

The participants were presented with various sounds through headphones. They were told that these sounds were caused by the impact of objects, and were instructed to categorize the material that was the source of the sound as wood, metal, or glass (three-alternative forced choice).

### Impact sounds

The non-reverberant sounds were the same as those used in a previous study^19^. First, the impact sound of 15 objects (5 wooden bars, 5 metal plates, and 5 glass vases) were modelled by the sum of exponentially decaying sine waves. The pitches of the sounds were adjusted to note C (an octave difference was allowed). We named these 15 sounds W_1_, …, W_5_, M_1_, …, M_5_, G_1_, …, and G_5_. W, M, and G represent wood, metal, and glass, respectively. The suffixes arbitrarily represent different objects within each material category and there are no meaningful associations between numbers across different material categories. Next, ambiguous sounds were generated as follows: W_1_, M_1_, G_1_, W_2_, M_2_, and G_2_ were placed on a circular continuum, and sound continua consisting of 22 steps between two adjacent sounds on the circle were created by morphing the decay rate and amplitude in each frequency component (Supplementary Fig. 4). The continuum consisted of 126 sounds: 6 original sounds +6 × 20 morphed sounds. Likewise, 126 sounds were included in the sound continuum that originated from W_3_, M_3_, G_3_, W_4_, M_4_, and G_4_, and 63 sounds originated from W_5_, M_5_, and G_5_. This procedure generated a total of 315 sounds^19^. It should be noted that for convenience the way of labelling the sounds was original to the present study. In the present study, we selected 210 of these 315 sounds to reduce the number of stimuli in order to test several conditions regarding reverberation and context within a practical amount of time allowed for the participants and experimenters. We selected sounds to be decimated on the basis of pilot experiments to maintain the overall shapes of the psychometric functions.

To apply a reverberation, the impact sounds were convolved with RIRs. We used 3 RIRs recorded in 3 different rooms^23^, resulting in sounds with 4 reverberation types (3 room reverberations and the non-reverberant original). The later portions of the convolved sounds were trimmed to fit to a duration of 2 s, and 0.5-s raised cosine ramps were applied to offset the sound.

The sounds were generated on a computer with a sampling frequency of 44.1 kHz and a 16-bit depth. They were presented diotically using an audio interface (UA-55, ROLAND), a headphone amplifier (M903, Grace Design), and headphones (HD595, Sennheiser). The amplifier gain was adjusted so that the peak amplitude (“fast” time weighted) of the presented sound recorded with an ear simulator was equal to that of the 1 kHz, 70 dB SPL tone.

### Experimental procedure

In the constant room condition, sounds with the same reverberation were presented repeatedly within one session. In the varying room condition, sounds with various types of reverberation were presented in random order. Care was taken to avoid two consecutive sounds having the same reverberation. In both conditions, 840 sounds (210 × 4 reverberation types) were presented once, divided into 8 sessions, each containing 105 sounds. The number of participants was balanced between those who started with sessions under the constant room condition and those who started under the varying room condition. Participants were instructed to report the material making the sound by pressing one of three buttons corresponding to the materials. Following the participant’s response, the next stimulus was presented after a time interval that was randomly chosen from [1.4, 1.6] s. We gave no explicit information to the participants prior to each session as to whether the session was under the constant room or varying room condition.

To avoid any gradual change in the participants’ identification criteria, training sessions were inserted prior to every session and at the beginning of the experiment. In the training sessions 15 non-reverberant original sounds (W_1_, M_1_, …, G_5_) were presented. The true material was visually fed back to the participant following his/her reaction. Sounds were presented 10 times in random order at the beginning of the experiment and twice at the beginning of each session. All the participants achieved a rate of more than 85% correct in the training at the beginning of the sessions.

### Estimation of probability of material selection

Estimating the probability of material selection from the participant’s responses is not trivial because sounds were presented only once in each of the constant and varying room conditions. In this study, first, the unnormalized probabilities *Q*(*x*|*s*) of each material *x* (wood, metal and glass) were estimated using the kernel smoothing method with an offset constant (1). They were then divided by the total to ensure that the total probability equalled 1 (2).

1 $$Q(x|s)=\sum _{R(s^{\prime} )=x}k(s,\,s^{\prime} )+\alpha .$$

2 $$P(x|s)=\frac{Q(x|s)}{{\sum }_{{x}^{^{\prime} }}Q({x}^{^{\prime} }|s)}.$$

Here, *R*(*s*) denotes the selected material following the presentation of the impact sound *s*, and *k*(*s*, *s*′) denotes the kernel. Since the sound continua are circular (Supplementary Fig. 4a), the variable *s* denoting the position in the continuum was circular in each of the circular continua. As the kernel we used a circular Gaussian kernel (or von Mises kernel).

3 $$k(s,s^{\prime} )=\exp (\frac{\cos (s-s^{\prime} )}{w}),$$

where *w* is a parameter determining the width of the kernel. The parameters *α* and *w* were determined in order to maximize the likelihood in a leave-one-out cross validation.

### Effect of reverberation

The effect of reverberation on each sound was defined by the distance between the probabilities for reverberant and non-reverberant sounds (4).

4 $$D({P}_{rev},{P}_{dry}|s)=\sqrt{\sum _{x}{(\sqrt{{P}_{rev}(x|s)}-\sqrt{{P}_{dry}(x|s)})}^{2},}$$

where *P*_*rev*_ and *P*_*dry*_ denote the probabilities for reverberant and non-reverberant sounds, respectively. We employed the Hellinger distance as the probability distance measure because it has several advantages over other distance measures: it satisfies metric axioms; and it is robust against small probabilities because it does not take logarithm or reciprocal.

Finally, the distances were averaged for all 3 rooms and 315 sounds to obtain the effect of reverberation on a given participant (5).

5 $${\rm{Effect}}\,{\rm{of}}\,{\rm{reverberation}}=\frac{1}{3}\sum _{rev}\frac{1}{315}\sum _{s}D({P}_{rev},{P}_{dry}|s).$$

The Hellinger distance was also used in multidimensional scaling to visualize inter- and intra-participant variability (Fig. 3b) and in quantifying the distance between the probabilities of different reverberation types (Fig. 3c). The average distance between the responses to non-reverberant and reverberant sounds was calculated with equation (5). The average distance between the responses to reverberant sounds in one room and those with another room was calculated with equation (6).

6 $${\rm{Ave}}.{\rm{distance}}\,{\rm{of}}\,{\rm{reverb}}.{\rm{vs}}.{\rm{reverb}}.=\frac{1}{3}\sum _{revA!=revB}\frac{1}{315}\sum _{s}D({P}_{revA},{P}_{revB}|s).$$

The confidence intervals (the 2.5 and 97.5 percentiles) of the distances were calculated by bootstrapping. The bootstrap distances were calculated from the material selection probabilities of the resampled stimuli. The set of resampled stimuli consisted of 15 pairs of sounds resampled from 15 pairs of sounds (W_1_ – M_1_, M_1_ – G_1_, G_1_ – W_2_, …, G_5_ – W_5_), and the morphed stimuli between those resampled pairs. Resampling was repeated 10,000 times with replacement.

### Linear model of material classification

Linear discriminant analysis (LDA) was used to model a participant’s responses with acoustic features (attack time, decay rate, spectral bandwidth, and spectral roughness) as independent variables and selected materials as dependent variables^19^. All the independent variables were converted into z-scores before being fed into the LDA model.

We defined the attack time as the time needed for the amplitude envelope to increase from 10% to 90% of its first peak value. The amplitude envelope was calculated by lowpass filtering the absolute values of a Hilbert-transformed waveform. The cutoff frequency of the lowpass filter was 50 Hz^19^.

The decay rate was defined by the negative of the slope of the logarithmic amplitude envelope, normalized by the spectrum centroid C (7).

7 $${\rm{C}}=\frac{{\sum }_{k}{s}_{k}{\omega }_{k}}{{\sum }_{k}{s}_{k}},$$

where *s*_*k*_ and *ω*_*k*_ denote the magnitude spectrum and the frequency, respectively. The slope was calculated using linear regression of the logarithmic amplitude envelope onto time. The regression was conducted in the interval from the peak of the envelope to the point where it decreased to 10% of the peak value.

The spectral bandwidth was calculated with equation (8).

8 $${\rm{spectral}}\,{\rm{band}}\,{\rm{width}}=\sqrt{\frac{{\sum }_{k}{s}_{k}{({\omega }_{k}-C)}^{2}}{{\sum }_{k}{s}_{k}}}.$$

The spectral roughness was calculated as the sum of the partial roughness (10) for all pairs of frequencies^19,29^.

9 $${\rm{spectral}}\,{\rm{roughness}}=\frac{1}{{a}^{0.2}}\sum _{m < n}{r}_{mn}.$$

10 $${r}_{mn}=0.5{(s{}_{m}{s}_{n})}^{0.1}{(\frac{2{\rm{\min }}({s}_{m},{s}_{n})}{{s}_{m}+{s}_{n}})}^{3.11}({e}^{-3.5{v}_{mn}}-{e}^{-5.75{v}_{mn}}).$$

11 $${{\rm{v}}}_{mn}=\frac{0.24\,({\omega }_{n}-{\omega }_{m})}{0.0207{\omega }_{m}+18.95}.$$

In the previous studies, the spectral roughness depended on the overall amplitude^26^. Specifically, it was proportional to the amplitude to the power of 0.2. In the current study, to make the spectral roughness independent of the overall amplitude, it was normalized by the root mean square of the sound to the power of 0.2, *a* ^0.2^ (9).

### Contribution of acoustic features to material identification

The contribution of the acoustic features was derived using the parameters of the LDA model. In LDA, a linear subspace is constructed in which the selected materials are most separated from one another. We defined the contribution of an acoustic feature as the sum of square of the loadings of the feature in the subspace divided by the dimension of the subspace (12).

12 $${C}_{f}=\frac{1}{d}\sum _{i=1}^{d}{w}_{if}^{2},$$

where *C*_*f*_ denotes the contribution of the feature *f*, and *w*_*if*_ denotes the loading of *f* in the *i*-th discriminant function. The variable *f* takes one of the four acoustic features. Since the loadings in LDA form an orthonormal system, the sum of the square of all the loadings in the subspace equals the dimension of the subspace $d={\sum }_{i,f}{w}_{if}^{2}$. In this study since there were three dependent variables to choose from, *d* = 3 − 1 = 2. The confidence intervals of the *C*_*f*_ were calculated by bootstrapping the stimuli as with the confidence intervals of the Hellinger distances.

### Statistical test

The differences between the effects of reverberation and the contribution of acoustic features under constant and varying room conditions were tested using the Wilcoxon signed rank test.

### Data availability

The datasets generated during and/or analysed during the current study are available from the corresponding author on reasonable request.

## Electronic supplementary material

Supplementary information
