# Supplementary material for: Context-Dependent Effect of Reverberation on Material Perception from Impact Sound
Source: Sci Rep. 2017 Nov 28;7:16455. doi: 10.1038/s41598-017-16651-4 (PMC5705663; doi:10.1038/s41598-017-16651-4)
Supplement: Supplementary file 1 — Supplementary information [file 41598_2017_16651_MOESM1_ESM.pdf]

Supplementary information

Article Title

Context-Dependent Effect of Reverberation on Material Perception from Impact Sound

Authors

Takuya Koumura and Shigeto Furukawa

Supplementary Figure 1

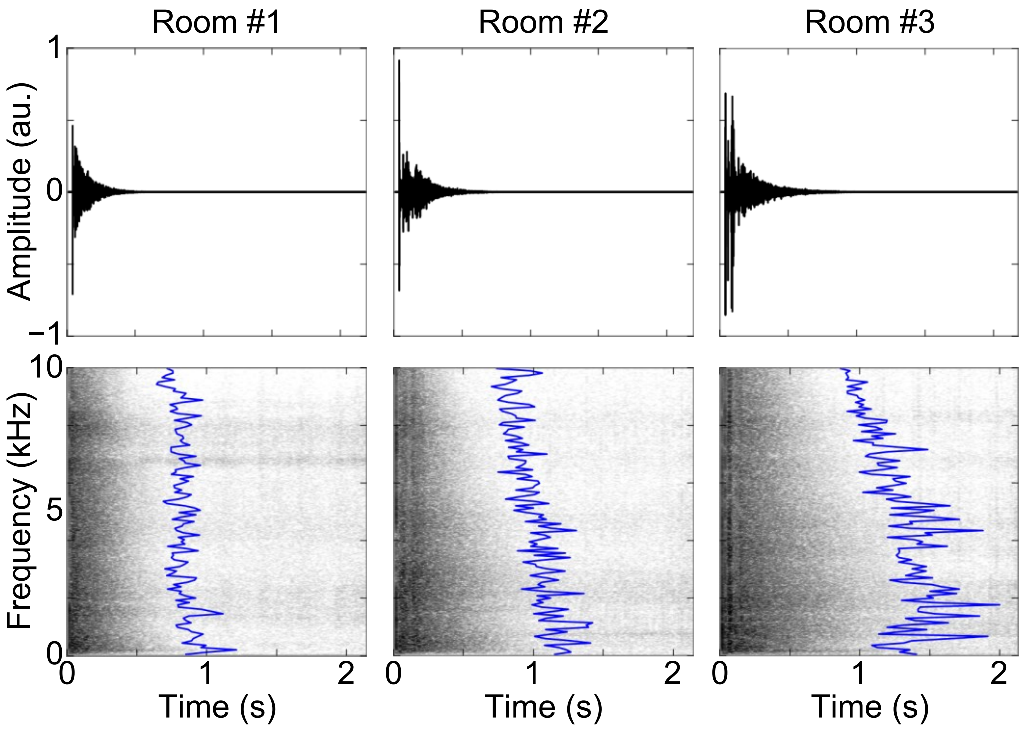

Room impulse responses.

Waveforms of three impulse responses (top) and the corresponding spectrograms (bottom). In the spectrograms, the time at which the amplitude envelope in each frequency band decreases by 60 dB (usually called RT60) is shown by the blue lines.

Supplementary Figure 2

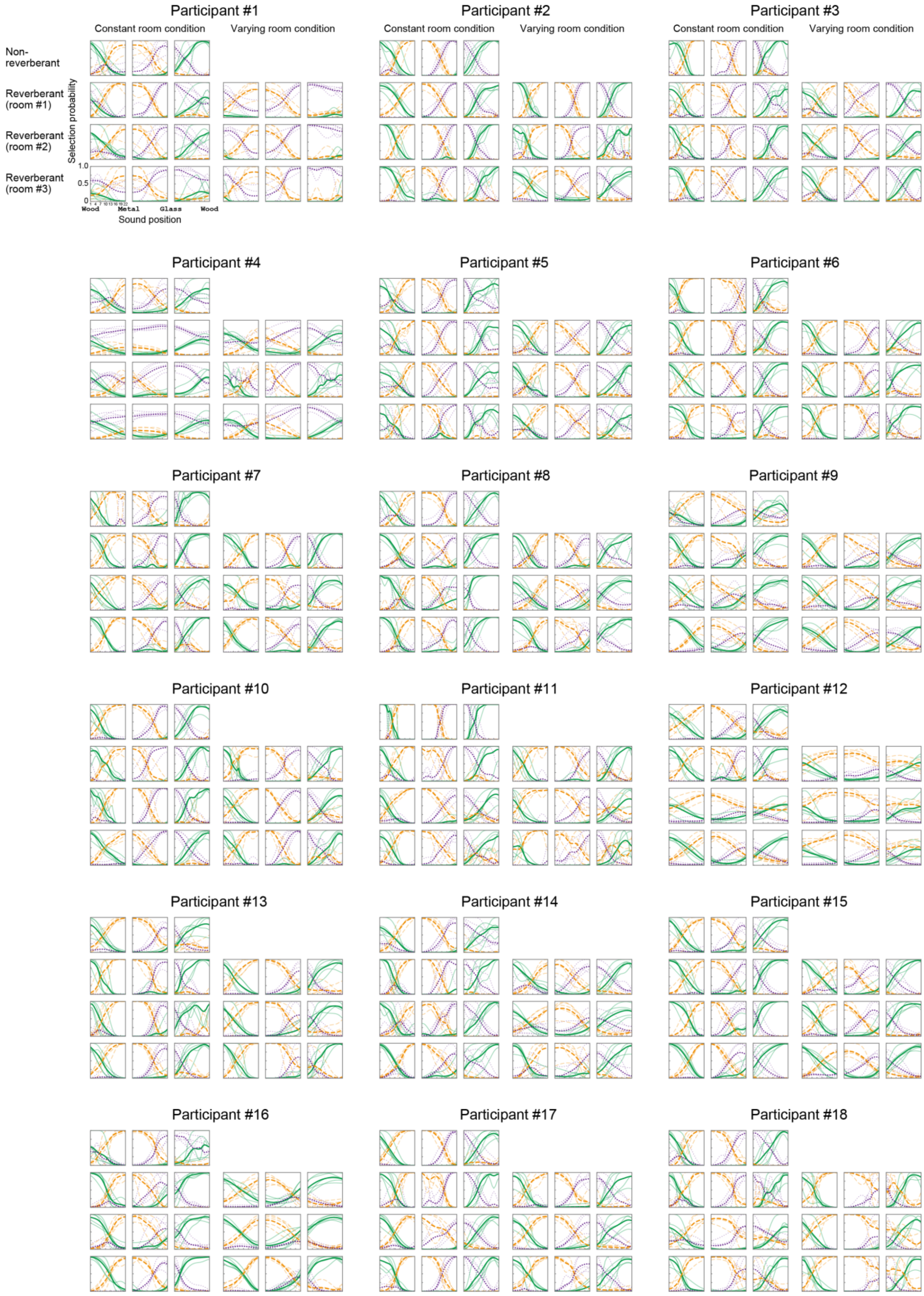

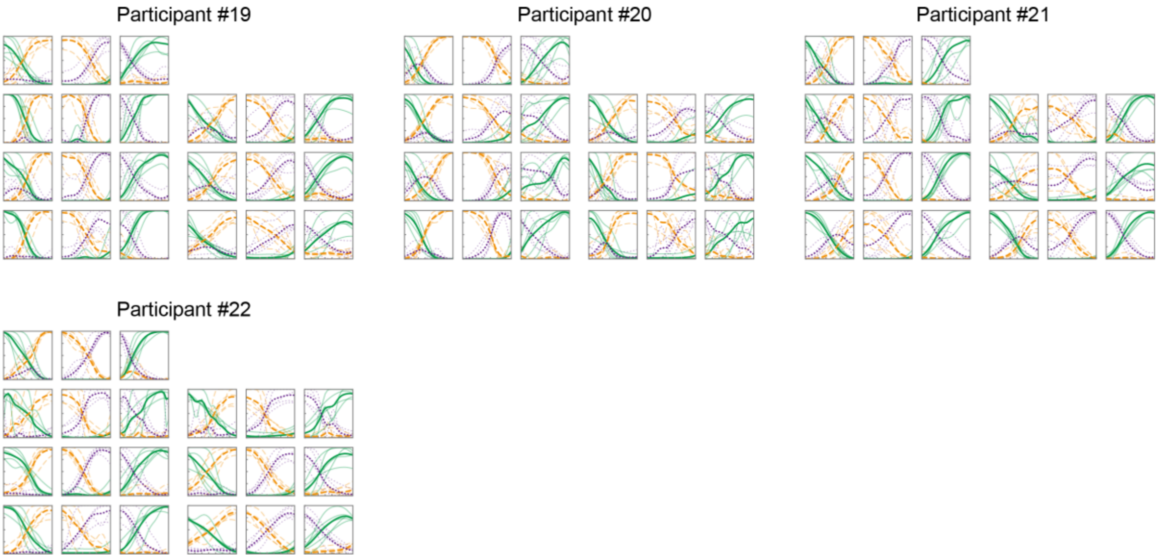

Material selection probabilities under all conditions for all participants.

With each participant, we show the probabilities of the selected materials for the non-reverberant sounds (top rows), the reverberant sounds in rooms #1 to #3 (second to fourth rows) under the constant room condition (left) and the varying room condition (right). The horizontal axis shows the sound positions in the sound continua between different materials. The thick dark lines indicate the average responses to all objects with the same material pairs. The thin light lines indicate the percentages of classification in each object pair. Solid green line: wood, dashed orange line: metal, dotted purple line: glass.

Supplementary Figure 3

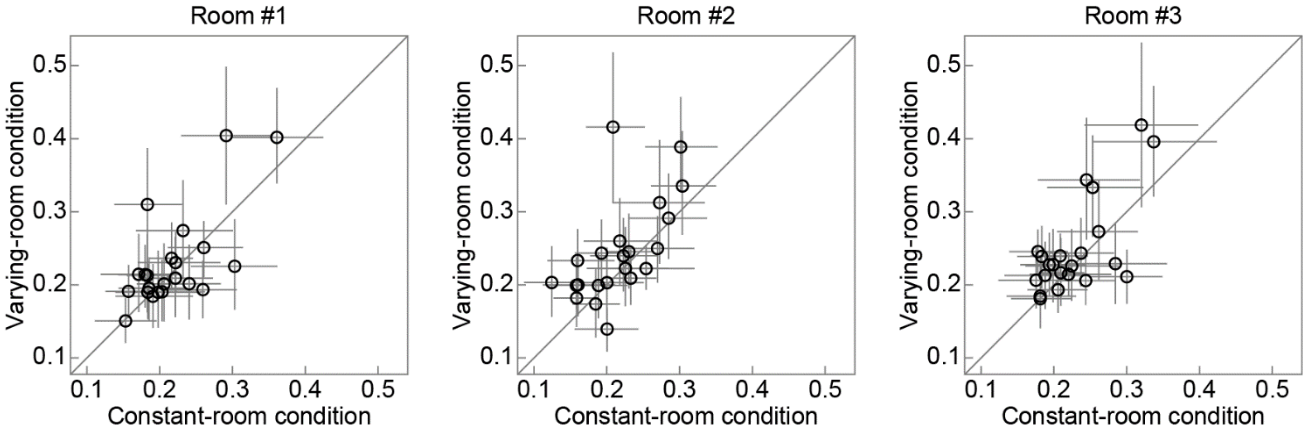

Effect of reverberation in each room.

Comparison of the size of the reverberation effect under the constant room condition (horizontal axis) and the varying room condition (vertical axis) in each room, before averaging over the rooms. Other conventions are the same as in Fig. 4. The size of the reverberation effect was similar across the rooms, suggesting that the context dependence of reverberation on material identification is not specific to certain RIRs.

Supplementary Figure 4

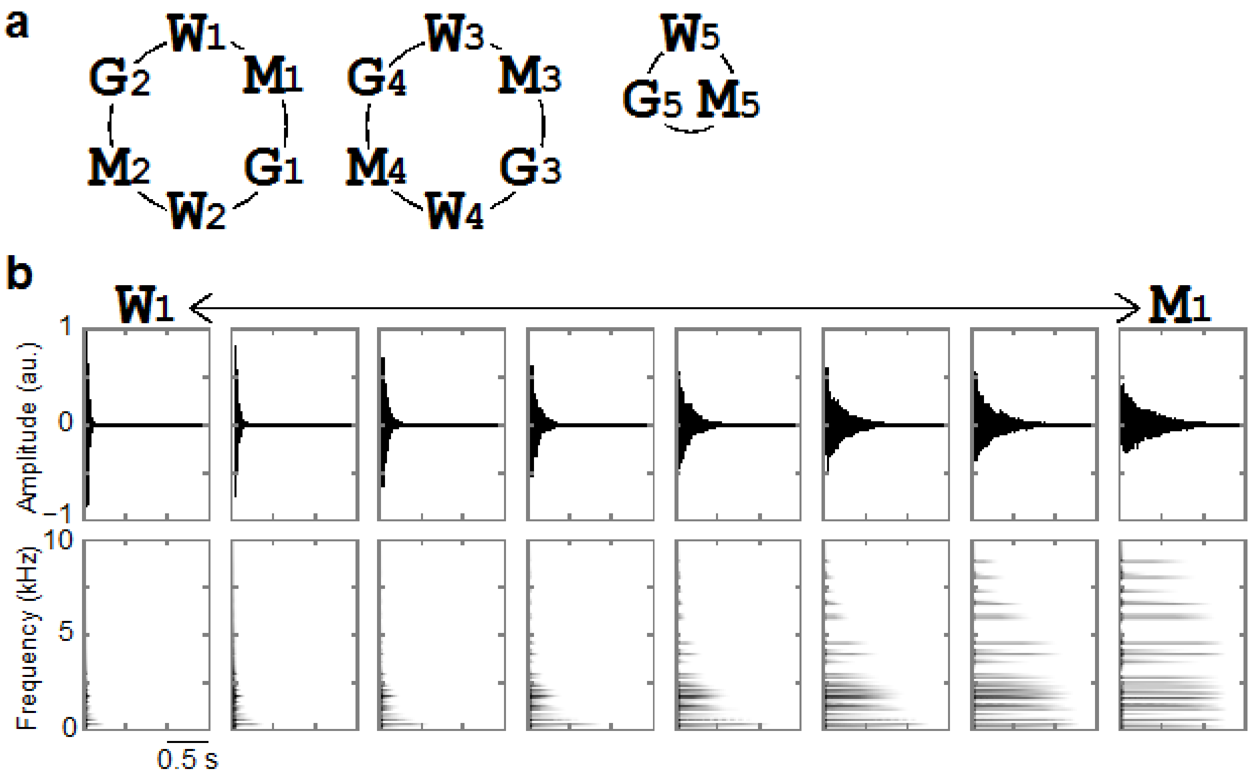

Sound continua.

(**a**) Original impact sounds (denoted as W*, M*, and G*) were placed on a circular sound continuum, and intermediate sounds between a pair of adjacent original sounds along the circle were synthesized by morphing. The sounds were provided by Aramaki et al[1]. W, M, and G represent wood, metal, and glass, respectively. Note that the suffix numbers arbitrarily represent different materials within each category and there is no meaningful association between numbers across different material classes. (**b**) Waveforms (top) and spectrograms (bottom) of the sounds on the continuum between W_1 and M_1. Eight sounds are shown as examples. The spectrum components and decay rate are morphed along the axis of the continuum. See Materials and Methods for details.

Supplementary Discussion 1

Consistent material identification

Our results indicated that reverberation altered material identification. However, a possible criticism is that the participants could not identify materials from a reverberant sound based on consistent criteria and this led to an apparently large effect of reverberation. To test whether participants identified materials in a consistent manner and not randomly, we computed the classification accuracy of the LDA model and compared it with the classification accuracy of the null model, which assumed random material identification. A thousand null models were constructed by shuffling the correspondence between independent and dependent variables, leading to 1000 null classification accuracies per participant and per context.

The classification accuracies for all the participants under both constant and varying room conditions were larger than those for all the null models. This result indicates that the participants identified materials based on some consistent criteria. The result also indicates that material identification by the participants can be described by a linear combination of acoustic features.

Supplementary Table 1

Specifications of rooms in which impulse responses were recorded.

	Volume (m^3)	Surface area (m^2)
Room #1	2,000	1,000
Room #2	5,700	2,500
Room #3	7,200	2,700

Three RIRs recorded in 3 different rooms were used in this study. The RIRs and room specifications were obtained from a database[2]

References

1. Aramaki, M., Besson, M., Kronland-Martinet, R. & Ystad, S. Controlling the perceived material in an impact sound synthesizer. *IEEE Trans. Audio, Speech Lang. Process.* **19,** 301–314 (2011).
2. Architectural Institute of Japan. *Sound Material in Living Environment*. (Gihodo Shuppan, 2004).
